# 3D Domain Swapping Causes Extensive Multimerisation of Human Interleukin-10 When Expressed *In Planta*

**DOI:** 10.1371/journal.pone.0046460

**Published:** 2012-10-01

**Authors:** Lotte B. Westerhof, Ruud H. P. Wilbers, Jan Roosien, Jan van de Velde, Aska Goverse, Jaap Bakker, Arjen Schots

**Affiliations:** Laboratory of Nematology, Wageningen University, Wageningen, The Netherlands; Cairo University, Egypt

## Abstract

Heterologous expression platforms of biopharmaceutical proteins have been significantly improved over the last decade. Further improvement can be established by examining the intrinsic properties of proteins. Interleukin-10 (IL-10) is an anti-inflammatory cytokine with a short half-life that plays an important role in re-establishing immune homeostasis. This homodimeric protein of 36 kDa has significant therapeutic potential to treat inflammatory and autoimmune diseases. In this study we show that the major production bottleneck of human IL-10 is not protein instability as previously suggested, but extensive multimerisation due to its intrinsic 3D domain swapping characteristic. Extensive multimerisation of human IL-10 could be visualised as granules *in planta*. On the other hand, mouse IL-10 hardly multimerised, which could be largely attributed to its glycosylation. By introducing a short glycine-serine-linker between the fourth and fifth alpha helix of human IL-10 a stable monomeric form of IL-10 (hIL-10^mono^) was created that no longer multimerised and increased yield up to 20-fold. However, hIL-10^mono^ no longer had the ability to reduce pro-inflammatory cytokine secretion from lipopolysaccharide-stimulated macrophages. Forcing dimerisation restored biological activity. This was achieved by fusing human IL-10^mono^ to the C-terminal end of constant domains 2 and 3 of human immunoglobulin A (Fcα), a natural dimer. Stable dimeric forms of IL-10, like Fcα-IL-10, may not only be a better format for improved production, but also a more suitable format for medical applications.

## Introduction

Recombinant DNA technology has revolutionized the production and application of pharmaceutical proteins. Current heterologous production hosts include bacteria, yeasts, insect and mammalian cells, and, more recently, plants. Most expression systems have been rapidly improved in terms of yield and cost efficiency in the last decades. However, these advances have mainly been achieved with antibodies and hormones, which are relatively stable proteins. Recently more attention in optimizing the production of biopharmaceutical proteins is directed to their intrinsic properties that result of post-translational modifications and folding processes. Improved insight in these processes may increase the yield of still poorly expressed proteins.

Many cytokines have a promising therapeutic potential. However, several cytokine families show a short half-life *in vivo* and are poorly expressed in heterologous hosts. Human interleukin-10 (IL-10) is such a cytokine that may be used for treatment of many inflammatory and autoimmune diseases due to its immunosuppressive properties [1], [2]. Generally, IL-10 facilitates the return of the immune system to homeostasis after clearance of antigen and plays an important role in conferring oral tolerance. It exerts its function through reduction of the activity of macrophages, inhibition of antigen presentation by dendritic cells and inhibition of the production of pro-inflammatory cytokines by antigen presenting cells and T lymphocytes [3]–[6]. The human IL-10 gene encodes a 178 amino acid protein including a N-terminal signal peptide for secretion. An IL-10 monomer consists of six alpha helices (A–F) with two internal disulphide bridges (Cys30-Cys126 and Cys80-Cys132). Two monomers are stabilized into a biologically active dimer by exchanging their C-terminal domains composed of the helices E and F, a process called 3D domain swapping [7]–[9].

Human interleukin-10 has previously been produced in bacterial systems for medical purposes [10], [11], and in insect and mammalian cells for research purposes. The use of plants as a production platform for IL-10 provides a cheap alternative compared to bacterial, insect and mammalian expression systems. As plants are eukaryotes they can correctly fold and assemble proteins, and are able to perform complex post-translational processes, such as glycosylation. Plants as production hosts for IL-10 offer an extra advantage as they have a low risk of contamination with human pathogens, especially relevant when producing immunosuppressive molecules for medical application. Human IL-10 was produced for the first time *in planta* by stable transformation of a low-alkaloid *Nicotiana tabacum* variety [12]. High transcript levels were contrasted by low protein levels with a maximum of 0.000069% of total soluble protein (TSP). Biological activity of plant-derived human IL-10 was shown *in vitro* and *in vivo* without the need for purification [12], [13]. Yield could be increased to 0.55% of TSP by transient expression of human IL-10 fused to an elastin-like polypeptide combined with retention in the endoplasmic reticulum (ER), but biological activity was not confirmed [14]. From these experiments it was concluded that protein instability is a major bottleneck for human IL-10 production.

We show that 3D domain swapping is an important bottleneck for human IL-10 production in *Nicotiana benthamiana*. Human IL-10 multimerises extensively as was visualised *in situ* using GFP fusions. Domain swapping could be prevented by engineering a stable monomer [15] that regained biological activity through fusion to the Fc portion of IgA, a natural dimer. Identification of this expression bottleneck enabled us to increase yield considerably to levels that approach the economic threshold.

## Results

### Yield of Mouse IL-10 is Significantly Higher Compared to Human IL-10

**Figure 1:**
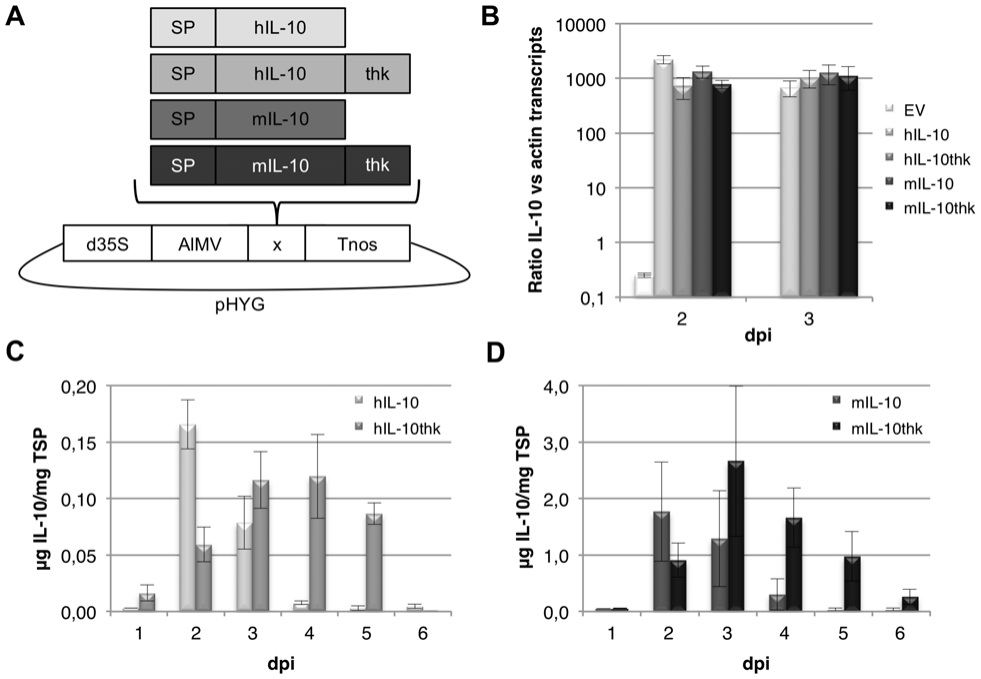
Expression data of human and mouse IL-10 in transiently transformed *Nicotiana benthamiana* leaves. Use of the thk-tag gives an increasing boost in yield for both human and mouse IL-10 from 2 days post infiltration (dpi). Strikingly, mouse IL-10 yield was significantly higher compared to human IL-10, regardless of ER-retention. Differences in yield could not be explained by differences in mRNA transcript levels. (A) Schematic representation of expression cassettes and vector used. Expressed genes include the native coding sequence of the human (h) or mouse (m) IL-10 gene including signal peptide for secretion (SP) with or without a 3′ tag coding for a thrombin cleavage site, a 6xHis-tag and the ER retention sequence KDEL (thk). All expression cassettes include the 35S promoter of the Cauliflower mosaic virus with duplicated enhancer (d35S), 5′ leader sequence of the Alfalfa mosaic virus RNA 4 (AlMV) and *Agrobacterium tumefaciens* nopaline synthase transcription terminator (Tnos). (B) Relative transcript levels of IL-10 versus actin as determined by Q-PCR on 2 and 3 dpi (*n* = 3, error bars indicate standard error). (C/D) Human and mouse IL-10 yield in crude extracts (1 to 6 dpi) in µg per mg total soluble protein (TSP) as determined by ELISA (*n* = 3, error bars indicate standard error).

To determine the maximum yield of human (h) and mouse (m) IL-10 in a transient expression system, leaves of 5–6 weeks-old *Nicotiana benthamiana* plants were agro-infiltrated. The expression vector contained the complete native coding sequence of the human or mouse IL-10 gene with or without a 3′ tag coding for a thrombin cleavage site, a 6xHis-tag and the ER retention sequence KDEL (thk) (Fig. 1a). Adequate transcription of the constructs was confirmed two and three days post infiltration (dpi) through determination of mRNA levels by means of quantitative PCR. Similar relative transcription levels (around 1000 transcripts of IL-10 per β-actin transcript) were found for all four constructs on both days (Fig. 1b). Human and mouse IL-10 yield was determined on 1–6 dpi using a sandwich ELISA (Fig. 1c and 1d). Maximum human and mouse IL-10 yield was obtained between 2–4 dpi. Although maximum yields were similar for native and ER-retained IL-10, expression levels of ER-retained IL-10 remained higher over time. Considering average yields from 2 to 5 dpi showed that ER-retained IL-10 resulted in 11 and 10-fold more protein for human and mouse IL-10 respectively. More striking was the observation that even though mRNA transcript levels were comparable, mouse IL-10 yield was significantly higher when compared to human IL-10 (*P* = 0.043), regardless of ER-retention (*P* = 0.003). The average yield of mIL-10 was 19 and 17-fold higher for secreted and ER-retained IL-10 respectively. Retention of a protein in the ER can lead to increased yield due to the presence of chaperones that assist protein folding and the absence of many proteases explaining reduced degradation over time [16]. However, as the difference in expression level between human and mouse IL-10 was significant despite ER-retention another factor than protein degradation supposedly influences yield.

### Human IL-10 Accumulates in Granules

**Figure 2:**
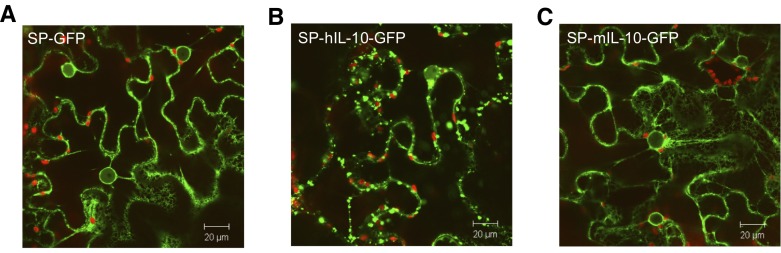
Whole mount confocal microscopy output of leaves expressing human or mouse IL-10 fused to GFP. Highly mobile globular granules of up to 5 µm in size were observed traveling along cytoplasmic and/or ER strands for SP-hIL-10-GFP only. (A) GFP preceded by the *Arabidopsis thaliana* chitinase signal peptide for secretion (SP-GFP). (B/C) The native open reading frame of human (h) and mouse (m) IL-10 including the native signal peptide (SP) with GFP fused C-terminally.

To investigate the cellular fate of human and mouse IL-10 *in planta*, GFP was fused C- and N-terminally to both proteins and expression was monitored by confocal microscopy. Plants expressing hIL-10-GFP showed fluorescent globular structures up to 5 µm in size (Fig. 2b). These granules resembled Golgi-bodies and were, regardless of their size, highly mobile as they travelled along ER strands. In contrast, mIL-10-GFP showed no or negligible signs of this phenomenon (Fig. 2c). For both proteins, fluorescence was observed in the nuclear envelope, the ER and, putatively, the apoplast as expected for a secretory protein (Fig. 2a). The results of C-terminal GFP fusions harbouring the native IL-10 signal peptides, like the unfused variants, are shown. N-terminal GFP fusions gave similar results (data not shown). Formation of granules may be caused by aggregation of folding intermediates or extensive multimerisation of human IL-10. Since mouse IL-10 did not show granules as observed for human IL-10, we assumed that formation of granules hinders yield of biologically active IL-10.

### Glycosylation of IL-10 Hinders Granulation

**Figure 3:**
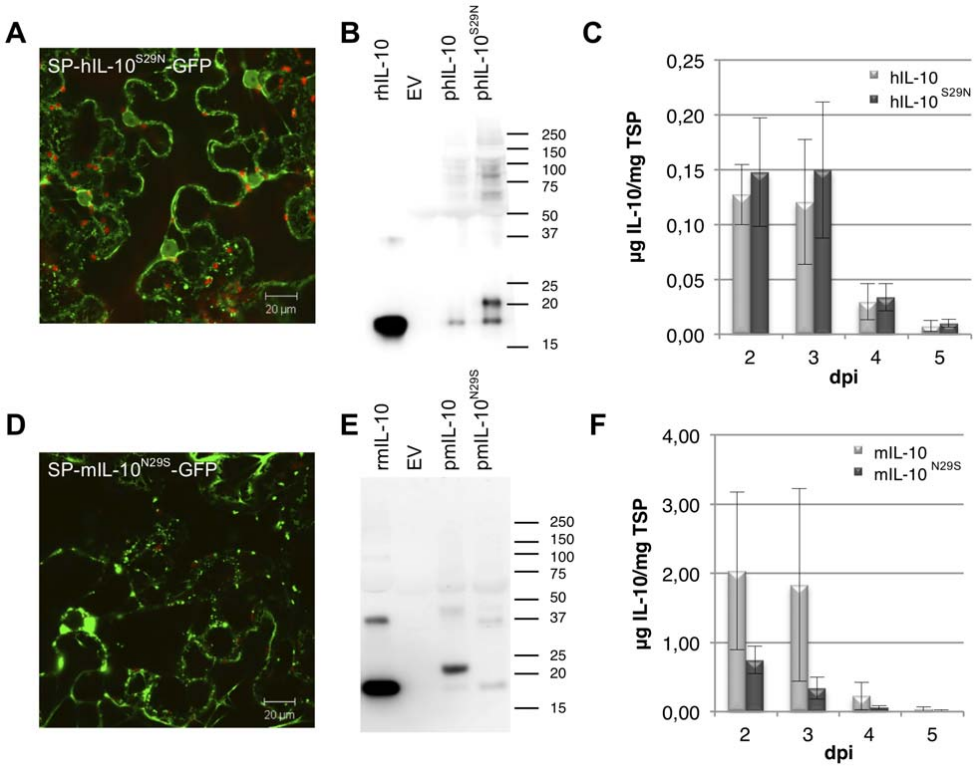
Analysis of the effect of *N*-glycosylation at Asn29 on granulation. Glycosylation of IL-10 plays a role in preventing granulation. (A/B) Whole mount confocal microscopy output of leaves expressing GFP fused C-terminally to human (h) and mouse (m) IL-10 including native signal peptide (SP) and with introduced (S29N) or removed (N29S) glycosylation site, respectively. (C/D) Western blot analysis under reducing conditions of plant produced (p) hIL-10 and mIL-10 with and without glycosylation site. As controls, empty vector (EV) and 50 ng recombinant (r) *E. coli* produced hL-10 and mIL-10 were used. A molecular weight marker is indicated in kDa. (E/F) Yield of hIL-10 and mIL-10 with and without glycosylation site in crude extracts 2 to 5 days post infiltration (dpi) as determined by ELISA (*n* = 4, error bars indicate standard error).

Human and mouse IL-10 have a homology of 73% at amino acid level. The most apparent difference between human and mouse IL-10 during post-translational processing is the glycosylation of mouse IL-10. Both proteins have one potential *N-*glycosylation site (Asn134) that is not glycosylated, while mouse IL-10 has yet another site that is glycosylated (Asn29). As glycosylation can stabilize a protein and influence protein folding by mediating interaction with chaperones, it may explain the difference in protein processing between human and mouse IL-10. To investigate the influence of glycosylation of IL-10 on granulation, the glycosylation site of mouse IL-10 was introduced in human IL-10 and removed from mouse IL-10. In both cases this was done by a single nucleotide mutation causing the serine of human IL-10 on position 29 to change into an asparagine and vice versa (hIL-10^S29N^ and mIL-10^N29S^). Figures 3a and 3d show a micrograph of GFP fused C-terminally to hIL-10^S29N^ and mIL-10^N29S^, respectively. Granulation of hIL-10^S29N^-GFP was greatly reduced, however, never completely absent. For mIL-10^N29S^-GFP granulation was induced, however, never reached the same extent as seen for hIL-10-GFP.

To confirm the presence of a *N*-glycan on hIL-10^S29N^ and the absence on mIL-10^N29S^, all non-GFP fused variants were analysed by Western blot (Fig. 3b and 3e). *E. coli* produced human and mouse IL-10, hence non-glycosylated, had the same molecular mass of 18 kDa when compared to plant-produced hIL-10 and mIL-10^N29S^. The molecular weight of mIL-10 and hIL-10^S29N^ was, as expected upon single *N*-glycosylation, approximately 1.5 kDa higher. Both mIL-10 and hIL-10^S29N^ samples also showed a small proportion of non-glycosylated IL-10.

The effect of glycosylation on human and mouse IL-10 yield was compared. ELISA indicated that removal of the glycosylation of mouse IL-10 decreased yield on average 4-fold, however, this was found not to be significant (Fig. 3c). Introducing a glycosylation site in human IL-10 did not lead to a yield increase (Fig. 3f), however, for hIL-10 the band intensities on Western blot (Fig. 3b) showed that an increased amount of glycosylated hIL-10 was extracted. This discrepancy might be explained by interference of the glycan on Asn-29 of hIL-10^S29N^ with the binding of the monoclonal antibodies used in ELISA.

### Glycosylation does not Influence Biological Activity

**Figure 4:**
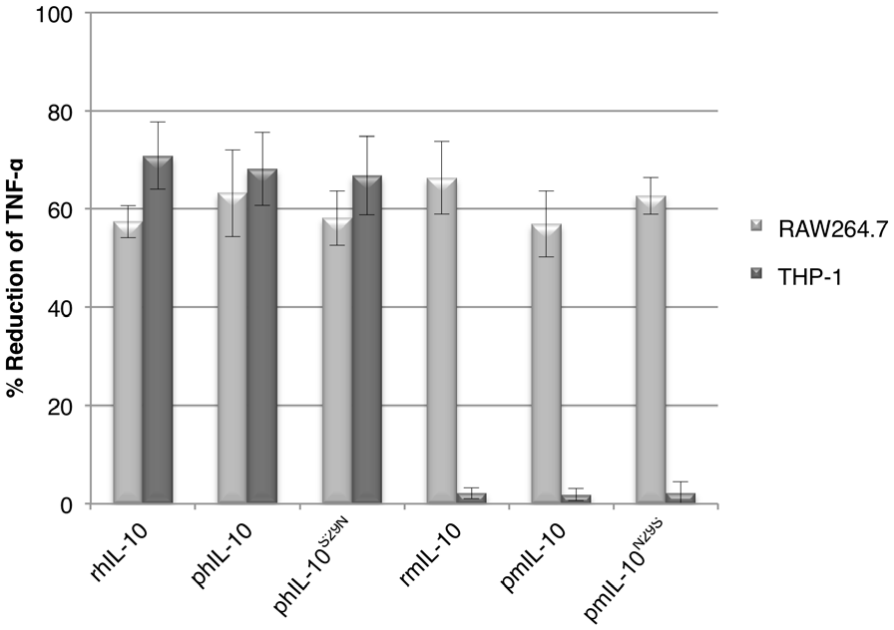
Biological activity of human and mouse IL-10 variants on human and mouse macrophages. Plant produced (p) and recombinant (r) *E. coli* produced human (h) or mouse (m) IL-10 were calibrated to contain the same amount of IL-10 as well as total soluble protein by using the empty vector control. Human (THP-1) and mouse (RAW264.7) macrophages were then pretreated with 10 ng/ml hIL-10 or mIL-10 for 20 min and subsequently stimulated with 1 µg/ml *E. coli* lipopolysaccharide. Tumor Necrosis Factor-alpha (TNF-α) expression was determined by ELISA and IL-10 activity is indicated as the percentage of inhibition of TNF-α expression as compared to the empty vector control (*n* = 3, error bars indicate standard error).

The possible effects of glycosylation on the biological activity of plant-produced human and mouse IL-10 was next assessed. The capacity of the different IL-10 variants to reduce Tumor Necrosis Factor-alpha (TNF-α) expression by human (THP-1) and mouse (RAW264.7) macrophages upon stimulation by lipopolysaccharide from *E. coli* was determined. Figure 4 shows the percentage inhibition of TNF-α secretion by macrophages when compared to the empty vector control. As expected, glycosylated as well as non-glycosylated mouse IL-10 suppressed the secretion of pro-inflammatory TNF-α from mouse macrophages, confirming that glycosylation is not necessary for mouse IL-10 activity [3]. Strikingly, our data show that glycosylated human IL-10 is as active as its native non-glycosylated form on both human and mouse cells. Apparently, the structure of human IL-10 is not negatively influenced by the glycosylation event. Neither non-glycosylated nor glycosylated mouse IL-10 was active on human cells.

### Granulation of Human IL-10 is Caused by Extensive Multimerisation

**Figure 5:**
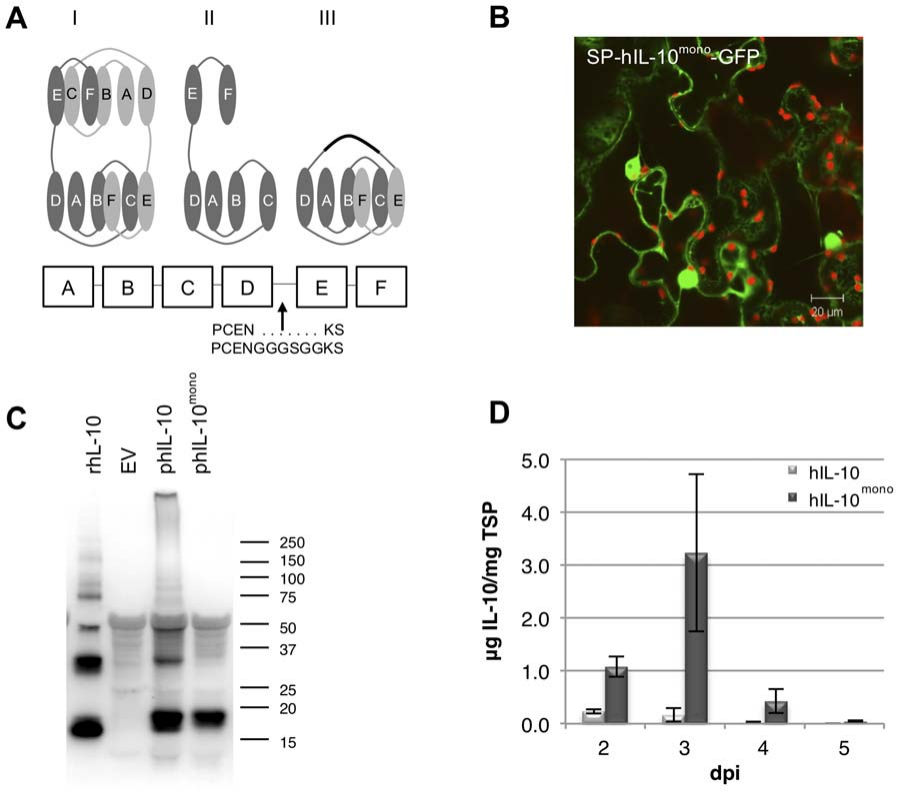
Analysis of expression of a stable monomeric form of human IL-10. A stable monomeric form of human IL-10 (hIL-10^mono^) does not granulate and yield increases 30-fold. (A) Three cartoons illustrating the human IL-10 (I) dimer, (II) monomer and (III) stable monomer structure, as well as a schematic representation of the human (h) IL-10 alpha helices A–F. Helices are represented by ovals, whereby a fragment of the amino acid sequence and the location of insertion of the small GS-linker is indicated. (B) Whole mount confocal microscopy output of GFP fused C-terminally to hIL-10^mono^ including native signal peptide (SP). (C) Western blot analysis under non-reducing conditions of plant produced hIL-10 and hIL-10^mono^. As controls, empty vector (EV) and 50 ng recombinant (r) *E. coli* produced hL-10 were used. A molecular weight marker is indicated in kDa. (D) Yield of hIL-10 and hIL-10^mono^ in crude extracts 2 to 5 days post infiltration as determined by ELISA (*n* = 3, error bars indicate standard error). Average yield of hIL-10^mono^ was significantly higher compared to hIL-10.

Literature describes IL-10 to be a 3D domain swapping protein, a term used to describe a process wherein two or more protein chains exchange identical structural elements or “domains” [9], [17]. Two IL-10 monomers dimerise by exchanging their E–F helices. However, when the concentration of a potentially 3D domain swapping protein is high, swapping of domains does not have to be limited to two partners [18]. For example, IL-10 can provide its A–D helices to one partner, while giving its E–F helices to another partner. This can create a chain reaction that can, in theory, continue until a stable form is reached. To determine if human IL-10 granulation was triggered by extensive multimerisation, a flexible linker was introduced between α-helices D and E of human IL-10. This allows helices E–F of one human IL-10 molecule to fold into its own A–D α-helices, creating a stable monomer (hIL-10^mono^) as designed by Josephson and co-workers [15]. Figure 5a shows three cartoons of the expected human IL-10 (I) dimer, (II) monomer and (III) stable monomer conformations. When the monomeric form of hIL-10 was fused to GFP no signs of granulation was observed (Fig. 5b). hIL-10^mono^-GFP was detected in the ER and, putatively, the apoplast. Unlike hIL-10-GFP and mIL-10-GFP, fluorescence was also observed in the cytoplasm and nucleoplasm. This may be indicative of partial failure of the protein to be taken up into the secretory pathway. However, on Western blot a band of 21 kDa, the expected size for hIL-10^mono^ with signal peptide, was never observed. Therefore, hIL-10^mono^-GFP must be expelled from the secretory pathway and enter the nucleoplasm by either diffusion or active transport. Active transport into the nucleus after expulsion from the ER was demonstrated with a secretory GFP fusion with the P-domain of the chaperone calreticulin [19].

Western blot analysis demonstrated that hIL-10^mono^ was indeed present as a monomer, whereas plant and *E. coli* produced hIL-10 also revealed bands corresponding to dimeric (36 kDa) and multimeric hIL-10 (Fig. 5c). In repetitive experiments a small proportion of dimeric hIL-10^mono^ was observed occasionally, but never larger multimers.

**Figure 6:**
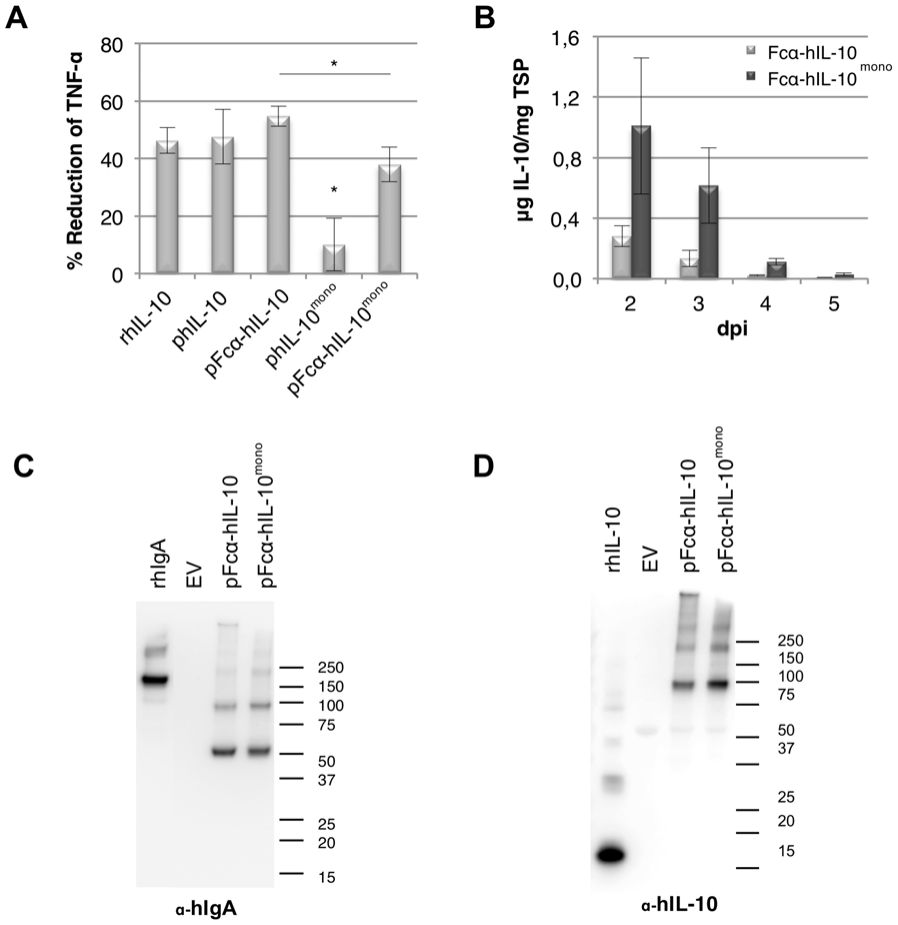
Analysis of biological activity and expression of human IL-10 and human IL-10^mono^ fused to Fcα. Forced dimerization of human IL-10^mono^ restores biological activity. (A) Bioactivity assay of hIL-10^mono^ and Fcα-hIL-10 fusion proteins on mouse macrophages (RAW267.4). Plant produced (p) and recombinant (r) *E. coli* produced hIL-10 were calibrated to contain the same amount of IL-10 as well as total soluble protein by using the empty vector control. Cells were then pretreated with 50 ng/ml hIL-10 for 20 min and subsequently stimulated with 1 µg/ml *E. coli* lipopolysaccharide. Tumor Necrosis Factor-alpha (TNF-α) expression was determined by ELISA and IL-10 activity is indicated as the percentage of inhibition of TNF-α expression as compared to the empty vector control (*n* = 4, error bars indicate standard error). Significant difference (*P*<0.05) between samples is indicated with an asterisk, where biological activity of hIL-10^mono^ was significantly different to all other samples. (B) Yield of human (h) IL-10 in crude extracts 2 to 5 days post infiltration as determined by ELISA (*n* = 3, error bars indicate standard error). (C/D) Western blot analysis under non-reducing conditions of plant produced (p) Fcα-hIL10 and Fcα-hIL-10^mono^ using an antibody raised against human IL-10 and human IgA for visualization, respectively. As controls, empty vector (EV), 50 ng recombinant (r) *E. coli* produced hIL-10 and 10 ng purified hIgA were used. A molecular weight marker is indicated in kDa.

Analysing average yields of hIL-10 and hIL-10^mono^ by ELISA revealed that the hIL-10^mono^ protein level was significantly higher (*P* = 0.048) resulting in 16-fold more protein. Maximum yield obtained with hIL-10^mono^ was 3,2 µg IL-10/mg (0.32%) TSP at 3 dpi (Fig. 5d). However, when hIL-10^mono^ was tested for biological activity by assessing its ability to suppress TNF-α expression by LPS stimulated macrophages, it appeared not to be functional (Fig. 6a), which was expected as IL-10 receptor binding studies suggest that the dimeric form of IL-10 confers biological activity [20]. This experiment was repeated several times and on some occasions slight activity of the hIL-10^mono^ was observed, however, always less than *E. coli* produced hIL-10. It appeared that the biological activity coincided with the proportion of dimeric hIL-10^mono^ seen on Western blot. It is likely that the extent of dimerisation and multimerisation of IL-10 is dependent on sample treatment such as freeze-thaw cycles, temperature and pH of the solution after extraction.

### Biological Activity of hIL-10^mono^ was Restored by Fusion to Fcα

To re-establish biological activity, dimerisation of hIL-10^mono^ was forced by fusion to the C-terminus of constant domains 2 and 3 of human immunoglobulin A2m1 (Fcα). As a reference, a construct with unmodified hIL-10 fused to Fcα was also made. Figure 6a shows the percentage inhibition of TNF-α secretion by macrophages when compared to the empty vector control. As mentioned before, hIL-10^mono^ was found to have reduced biologically activity, but by fusion to Fcα we were able to restore its biological activity (*P* = 0.0230). Also, fusion of native hIL-10 to Fcα slightly increased biological activity, but not significant. While the activity of the monomeric IL-10 Fcα fusion had no significant difference with *E. coli* or plant produced native hIL-10, a significant difference was found with the native IL-10 Fcα fusion (*P* = 0.0375).

Both fusion proteins showed similar accumulation patterns as observed for the unfused variants of IL-10. However, yield of Fcα-hIL-10^mono^ was slightly lower compared to the unfused variant on dpi 3, but was found not to be significant (Fig. 6b). This indicates that fusion with a stable fusion partner does not ensure increase in yield of IL-10, as was the case with fusion to elastin-like polypeptides [14], [21].

Western blot analysis using antibodies raised against hIL-10 showed that the largest proportion had the expected size of dimeric Fcα-hIL-10 (95 kDa) for both hIL-10 and hIL-10^mono^ fusions, but also bands at 200 kDa and 300 kDa were observed, which could again indicate the presence of multimers of these fusion proteins (Fig. 6c). Using antibodies raised against IgA revealed, in addition to all bands corresponding with the IL-10 blot, an extra band around 50 kDa (Fig. 6d). Because this band does not appear on the IL-10 blot, it is probably a cleavage product whereby IL-10 is removed. Specifically, a dimeric Fcα protein without hIL-10 would have an expected size of 50 kDa. Yet, no separated IL-10, expected at 18 or 36 kDa for monomeric and dimeric IL-10 respectively, was seen and must therefore be completely degraded.

## Discussion

Here we show that extensive multimerisation of human IL-10 is the most important factor limiting yield and not protein instability. Human IL-10 has previously been expressed in plants [12], [14], [21]–[23]. The highest yield of 0.55% of TSP was obtained by transient expression of human IL-10 fused to an elastin-like polypeptide while retained in the ER. It was suggested that retention in the protein friendly environment of the ER protects the protein against degradation and that elastin-like polypeptides might prevent aspecific aggregation by association with chaperones [12], [14]. These data suggested a role for protein instability or inefficient post-translational processing as a limiting factor for production of human IL-10**.** Protein instability as a bottleneck for production of IL-10 would not be surprising, as cytokines are known for their short half-life *in vivo*.

When we studied the cellular fate of human and mouse IL-10 *in planta* using GFP fusions, we revealed that human IL-10 accumulates in granules, while mouse IL-10 does not. The most evident difference between human and mouse IL-10 is the *N*-glycosylation of mouse IL-10. Both proteins have one potential *N-*glycosylation site (Asn134) that is not glycosylated, while mouse IL-10 has yet another site that is glycosylated (Asn29). Removal of the Asn29 glycosylation site resulted in increased granulation of mouse IL-10, whereas introduction of this glycosylation site in human IL-10 prevented granulation to a large extent. As glycosylation can aid in protein folding and stabilization, we hypothesized that the granules were in fact very large aggregates, potentially compartmentalised as a cell protective mechanism.

Aggregation can be due to protein instability and/or misfolding resulting in random hydrophobic patch interaction or, in the case of IL-10, due to extensive multimerisation. Literature describes IL-10 to be a 3D domain swapping protein, a term used to describe a process wherein two or more protein chains exchange identical structural elements or “domains” [9], [17]. IL-10 monomers form biologically active dimers by exchanging their E–F helices. However, at high concentrations of IL-10 it would be possible that the domain swapping of IL-10 is not limited to two partners and causes a chain reaction that, in theory, could go on unlimited until a stable form is reached. To determine whether human IL-10 granulation was due to protein instability or due to 3D domain swapping, the behaviour of a previously developed stable monomeric form of human IL-10 [15] was studied *in planta*. This stable monomeric form of human IL-10 has a short glycine-serine (GS)-linker between helices D and E allowing the helices E–F to fold back into its own hydrophobic core created by the A–D helices (human IL-10^mono^). No granulation was observed when human IL-10^mono^ was fused with GFP. Multimeric or high molecular weight bands of human IL-10^mono^ were also not detected by Western blot. Furthermore, yield was increased on average 16-fold by this minor modification of the human IL-10 protein. This expression level was comparable to the highest yield obtained for human IL-10 so far, however, without retention in the ER or fusion with a stable protein partner. Taken together, we show that not protein instability, but extensive multimerisation of human IL-10, due to its intrinsic 3D domain swapping characteristic, is the major limiting factor for yield.

Bennett and colleagues introduced the term 3D domain swapping [17] and up to date almost 300 proteins have been reported with 3D domain swapping ability [24]. Besides IL-10, the cytokines Granulocyte Macrophage Colony-Stimulating Factor (GM-CSF), Interleukin-5 (IL-5) and Interfernon-beta (IFN-β) are also described as 3D domain swapping proteins [9] and have been heterologously expressed in several platforms. Upon expression in *E. coli* the formation of inclusion bodies were described for these four cytokines [25]–[28]. Although formation of inclusion bodies could be indicative of multimerisation, they are a relatively common feature when expressing eukaryotic proteins in *E. coli* due to aggregation of folding intermediates. However, in the case of IFN-β and IL-5, experiments also demonstrated the need for slow removal of denaturing agents to prevent aggregation to reoccur after solubilisation of inclusion bodies [25], [26]. To our knowledge, IL-5 has not been produced in plants before and expression of human IFN-β in lettuce leaves resulted in poor yields [29]. On the other hand, many research groups have produced human GM-CSF *in planta*. Interestingly, human GM-CSF secreted by a tobacco cell culture could be stabilized in the growth medium by addition of bovine serum albumin, gelatin or salt, all methods that could inhibit protein aggregation [30], [31]. Next to that, when produced in rice seeds a morphological change in both plant protein body type I and the ER were observed together with high molecular weight variants of human GM-CSF from 50 to 120 kDa [32], [33]. Menassa and colleagues also described a 200 kDa weight variant of human IL-10 when expressed stably in a tobacco species [12]. For both human IL-10 and human GM-CSF yield from rice seeds was relatively high compared to the leaf-based and cell culture systems, however, extraction from rice seeds was only efficient using reducing agents [23], [34]. Unfortunately, use of reducing agents during extraction would increase production costs again, as the protein would need to be chemically refolded. There are many indications that extensive multimerisation has also hampered the production of other 3D domain swapping cytokines, and may represent a more common mechanism affecting yield than thus far anticipated. In addition, it is unclear how 3D domain swapping influences biopharmaceutical proteins during formulation, storage and administration.

Although we could increase the yield of human IL-10 significantly by expression of a stable monomeric form of IL-10, this form was not biologically active, as determined by its ability to reduce TNF-α secretion by lipopolysaccharide-stimulated macrophages. Even though human IL-10^mono^ has all the receptor binding sites, the affinity may be reduced. Josephson and co-workers did show biological activity of human IL-10^mono^ based on its ability to induce proliferation of a B cell line, however, a 10-fold higher amount of human IL-10^mono^ was needed to obtain the same effect as recombinant human IL-10 [15]. Stable monomeric forms of IL-5 were also created and shown to have reduced receptor affinity. Nevertheless, they did show biological activity with concentration dependence close to that of the wild type [35]. Such stabilized biologically active forms of 3D domain swapping cytokines will ensure that the protein stays in its biologically active conformation, which may have great benefit for formulation and administration to patients.

To mimic the natural dimerization of IL-10 while still preventing multimerisation, human IL-10^mono^ was fused to the naturally dimerising Fc portion of IgA2m1 (Fcα), which restored the ability to suppress TNF-α in stimulated macrophages. As well as ensuring dimeric conformation, it is likely that fusion with Fcα increases *in vivo* half-live of IL-10, which could be beneficial for efficacy. Our construct is comparable to that of mouse IL-10 fused to the N-terminus of a non-cytolytic version of the Fc portion of mouse IgG2a, which was shown to be functional *in vivo* with a prolonged efficacy due to its increased circulating half-life [36]. However, an increased half-life of IL-10 could also lead to unwanted systemic side effects, such as a compromised immune system. By using complete antibodies it may be possible to circumvent these side effects. Through the antigen binding capacity of an antibody with IL-10 fused C-terminally, specific cells or tissues can be targeted enabling IL-10 therapy for a variety of diseases, as was already demonstrated by Schwager and co-workers [37]. In disease therapy, use of a human fusion protein is desired to limit the chance of an immune response against the therapeutic agent. Also, in inflammatory disease therapy the use of IgA may be preferred over other antibody isotypes, as it does not activate the classical complement pathway. Thus, a stable molecule that ensures a biologically active conformation and targeting, such as Fcα-IL-10, may be a more suitable format for medical applications and may prove effective in therapy of inflammatory diseases.

## Experimental Procedures

### Construction of Expression Cassettes and Vector

The complete native open reading frames (ORF) of human and mouse IL-10 were amplified from the MegaMan™ Human Transcriptome cDNA library (Stratagene) and FirstChoice™ PCR-Ready Mouse Spleen cDNA library (Ambion), respectively, using oligonucleotides indicated in Table 1. Sense and antisense oligonucleotides included NcoI and KpnI restriction sequences, respectively, for subsequent cloning steps. Introduction of the NcoI restriction site resulted in the addition of two extra amino acids to the signal peptide at the N-terminus. All oligonucleotides used for subsequent reamplification, mutagenesis and insertion can be found in Table 1.

**Table 1:** Oligonucleotides used for construct re-amplification, mutagenesis and insertion.

Gene fragment	Function	*5' –>3'* Sense oligonucleotides	*5' –>3'* Antisense oligonucleotides
hIL-10	Isolation hIL-10 NcoI/KpnI	ccatgggcATGCACAGCTCAGCACTGCTCT	cggtaccTCAGTTTCGTATCTTCATTGTCA
mIL-10	Isolation mIL-10 NcoI/KpnI	ccatggccATGCCTGGCTCAGCACTGCTAT	ccggtaccTTAGCTTTTCATTTTGATCATC
pHYG adaptor	MCS including AscI/PacI	aattcggcgcgcctacgcgtaaggacgagctctgaggtacctctagattaattaaa	agcttttaattaatctagaggtacctcagagctcgtccttacgcgtaggcgcgccg
hIL-10-NotI	Removal stop codon,addition NotI	ccatgggcATGCACAGCTCAGCACTGCTCT	ggcggccgcGTTTCGTATCTTCATTGTCATG
mIL-10-NotI	Removal stop codon,addition NotI	ccatggccATGCCTGGCTCAGCACTGCTAT	ggcggccgcGCTTTTCATTTTGATCATCATG
Thk	Thrombin, 6xHIS, KDEL	ggccgcattagttcctcgtggttctgctagccatcaccatcaccatcacaaagatgagctatgacgtacgggtac	ccgtacgtcatagctcatctttgtgatggtgatggtgatggctagcagaaccacgaggaactaatgc
eGFP	Isolation GFP NcoI/KpnI	gtcgacggatccATGGTGAGCAAGGGCGAGGAGCTGTTC	aggtaccTTAGCTCATGACTGACTTGTAGAGCTCGTCCATGCCGAGAG
NotI-NcoI	Linker C-terminal GFP fusion	ggccgctgcagtcgacggatc	Catggatccgtcgactgcagc
mIL-10^N29S^	Removal glycosylationsite	GCCGGGAAGACAATAgCTGCACCCACTTCCC	GGGAAGTGGGTGCAGcTATTGTCTTCCCGGC
hIL-10^S29N^	Introduction glycosylationsite	CCCAGTCTGAGAACAaCTGCACCCACTTCCCAG	CTGGGAAGTGGGTGCAGtTGTTCTCAGACTGGG
hIL-10^mono^	Insertion GGGSGG-linker	AAACggtggcggatctgggggtAAGAGCAAGGCCGTGGAGCAGGTGAA	CTTacccccagatccgccaccGTTTTCACAGGGAAGAAATCGATGA
SpeI-hIL-10	Removal SP, addition SpeI	accatggactagtAGCCCAGGCCAGGGCAC	cggtaccTCAGTTTCGTATCTTCATTGTCA
h/m IL-10	Q-PCR analysis	ATGATCCAGTTTTACCTGG	AGAAATCGATGACAGCG
Nb β-actin	Q-PCR analysis	CCAGGTATTGCCGATAGAATG	GAGGGAAGCCAAGATAGAGC

Native sequences in capitals, added/mutated sequences in small and restriction sites are underlined. h; human, IL-10; interleukin-10, MCS; multiple cloning site, m; mouse, Nb; *Nicotiana* benthamiana, SP; signal peptide for secretion, thk; thrombin-6xHIS-KDEL tag.

To create hIL-10thk and mIL-10thk, both hIL-10 and mIL-10 ORFs were reamplified, resulting in the removal of the stop codon and replacement of the KpnI by a NotI restrictrion site. The thk coding DNA fragment could be added in frame. To remove and introduce the glycosylation site of mouse and human IL-10 respectively, the QuickChange XL kit (Stratagene) was used. For insertion of the 6 amino acid glycine-serine spacer overlap extension PCR was performed in combination with original hIL-10 oligonucleotides.

The DNA fragment encoding the enhanced green fluorescent protein (eGFP) was reamplified from gateway vector pK7FWG2 [38]. Constructs with eGFP fused C-terminally to IL-10 were composed of the IL-10 fragments ending with NotI (as described for the h/mIL-10thk constructs) the “NotI-NcoI” oligo and the eGFP fragment.

The constant domains 2 and 3 of human immunoglobulin A2m1 heavy chain including a N-terminal IgA signal peptide and a C-terminal linker sequence was synthetically constructed by GeneArt and fused N-terminally to reamplified SpeI-hIL-10 and SpeI-hIL-10mono fragments.

All constructs were placed under the control of the 35S promoter of the *Cauliflower mosaic virus* with duplicated enhancer (d35S) and the *Agrobacterium tumefaciens* nopaline synthase transcription terminator (Tnos). A 5′ leader sequence of the Alfalfa mosaic virus RNA 4 (AlMV) was included between the promoter and construct to boost translation. All elements were present in pRAP35 (or pUCAP35S [39]) from which expression cassettes were digested with AscI and PacI and ligated into a modified version of the expression vector pMDC32 [40], renamed pHYG. The pHYG vector resulted upon removal of the Gateway recombination sequences by digestion with EcoRI and HindIII and replacement with a DNA fragment (oligos “pHYG adaptor”) including AscI and PacI restriction sites for insertion of the expression cassettes.

All construct sequences were confirmed by sequencing (BaseClear) at the expression vector stage. The expression vectors were subsequently transformed to *Agrobacterium tumefaciens* strain MOG101 for plant expression.

### *Agrobacterium tumefaciens* Transient Transformation Assay

*Agrobacterium tumefaciens* clones were cultured overnight (o/n) at 28°C in LB medium (10g/l pepton140, 5g/l yeast extract, 10g/l NaCl with pH 7.0) containing 50 µg/ml kanamycin and 20 µg/ml rifampicin. The optical density (OD) of the o/n cultures was measured at 600 nm and used to inoculate 50 ml of LB medium containing 200 µM acetosyringone and 50 µg/ml kanamycin with x µl of culture using the following formula: x = 80000/(1028*OD). OD was measured again after 16 hours and the bacterial cultures were centrifuged for 15 min at 2800×*g*. The bacteria were resuspended in MMA infiltration medium (20g/l sucrose, 5g/l MS-salts, 1.95g/l MES, pH5.6) containing 200 µM acetosyringone till an OD of 1 was reached. After 1–2 hours incubation at room temperature, the two youngest fully expanded leaves of 5–6 weeks old *Nicotiana benthamiana* plants were infiltrated completely. Infiltration was performed by injecting the *Agrobacterium* suspension into a *Nicotiana benthamiana* leaf at the abaxial side using a 1 ml syringe. Infiltrated plants were maintained in a controlled greenhouse compartment (UNIFARM, Wageningen) and infiltrated leaves were harvested at selected time points.

### Total Soluble Protein Extraction

Leaves were immediately snap-frozen upon harvesting, homogenized in liquid nitrogen and stored at −20°C until use. Homogenized plant material was ground in ice-cold extraction buffer (50 mM phosphate-buffered saline (PBS) pH = 7.4, 100 mM NaCl, 10 mM ethylenediaminetetraacetic acid (EDTA), 0.1% v/v Tween-20, 2% w/v immobilized polyvinylpolypyrrolidone (PVPP)) using 2 ml/g fresh weight. Crude extract was clarified by centrifugation at 16.000×*g* for 5 min at 4°C and supernatant was directly used in an ELISA and BCA protein assay. For western blotting and biological activity assays, above-mentioned protein extraction would be followed by desalting using a G25 Sephadex column and filter sterilization (0.22 µm; Millipore Corporation).

### Quantification of Human and Mouse IL-10 mRNA Levels

mRNA was isolated from homogenized plant material using the RNAeasy Plant Mini Kit (Qiagen) according to suppliers protocol. A Turbo DNaseI (Ambion) treatment was included to remove any residual DNA. cDNA was synthesised using the SuperScript®III First-Strand Synthesis System (invitrogen) according to suppliers protocol. Samples were analysed by quantitative PCR in triplo using ABsolute SYBR Green Fluorescein mix (Thermo Scientific). *Nicotiana benthamiana* β-actin was used as a reference gene. Oligonucleotides used can be found in Table 1. Relative transcript levels of IL-10 versus actin were determined by the Pfaffl method [41].

### Quantification of Human and Mouse IL-10 Protein Levels

IL-10 protein concentration in crude plant extract was determined by ELISA. Human and mouse IL-10 ELISA Ready-SET-Go!® kits (eBioscience) were used according to suppliers protocol using the model 680 plate reader (BioRad) to measure the OD at 450 nm with correction filter of 690 nm. For sample comparison the total soluble protein (TSP) concentration was determined by the BCA method (Pierce) according to supplier’s protocol using bovine serum albumin (BSA) as a standard.

### Protein Analysis by Western Blot

Soluble plant proteins were separated under reducing or non-reducing conditions by SDS-PAGE on a NuPAGE® 12% Bis-Tris gel (Invitrogen). Recombinant *E. coli* produced human or mouse IL-10 (R&D Systems) or IgA1 (Invivogen) was used as a control. Proteins were transferred to an Invitrolon™ PVDF membrane (Invitrogen) by semi-dry blotting procedure. Thereafter the membrane was blocked in PBST-BL (PBS containing 0.1% v/v Tween-20 and 5% w/v non-fat dry milk powder) for 1 hour at room temperature, followed by overnight incubation with hIL-10 specific goat polyclonal antibody (R&D systems), mIL-10 specific rat monoclonal antibody (BioLegend) or IgA specific HRP conjugated goat polyclonal antibody (Sigma) in PBST at 4°C. The membrane was washed 5 times with 5 min intervals in PBST. For h/mIL-10 specific western blots the procedure was continued with a 1 hour incubation at room temperature with HRP conjugated secondary antibodies (Jackson ImmunoResearch) in PBST and washed again as described before. Finally, the SuperSignal West Femto substrate (Pierce) was used to detect HRP-conjugated antibodies.

### Confocal Microscopy

Plants were agro-infiltrated with expression cassettes encoding C-terminal fusions of GFP to human and mouse IL-10 as described previously. Leaves were taken from the plant and small sections were examined from the abaxial side using a Zeiss LSM510 confocal laser-scanning microscope in combination with an argon ion laser supplying a 488 nm wavelength.

### Biological Activity Assay

The monocyte THP-1 and monocyte/macrophage RAW264.7 cell lines were purchased from the American Type Culture Collection and cells were maintained at 37°C with 5% CO_2._ THP-1 cells were cultured in RPMI-1640 medium containing containing 4 mM L-glutamine, 25 mM HEPES and supplemented with 10% fetal calf serum, 50 U/ml penicillin and 50 µg/ml streptomycin. THP-1 monocytes were differentiated into macrophages for 4 days using 30 ng/ml PMA at a density of 3×10^5^ cells/ml in 96 well plates. Cells were allowed to rest for 2–3 days in medium without PMA prior to bioassays. RAW264.7 cells were cultured in DMEM containing 4 mM L-glutamine, 25 mM HEPES and supplemented with 10% newborn calf serum, 50 U/ml penicillin and 50 µg/ml streptomycin. Cells were sub-cultured every 2–3 days, whereby the cells were harvested by gently disrupting the monolayer with a cell scraper. For cell-based assays cells were seeded in 96 well plates at a density of 5×10^4^ cells/well and allowed to rest overnight. For bioassays cells were pre-treated with 10–50 ng/ml plant produced or recombinant *E. coli* produced human or mouse IL-10 (R&D Systems) in plant extract for 20 min, and subsequently stimulated with 1 µg/ml of lipopolysaccharide (Sigma). After overnight incubation supernatants were analysed with the mouse TNF-α ELISA Ready-Set-Go! Kit (eBioscience) according to the supplier’s protocol.

### Data Analysis

All data shown in the figures indicate the average of at least three biological replicates (*n*) that were determined by at least three technical replicates. In the figure legends *n* is indicated and error bars indicate the standard error. Significant differences between samples were calculated using the student’s t-test and regarded as significant when *P*<0.05. Significant differences in expression levels between constructs were calculated by using the whole data set from dpi 2 to 5 and are indicated in the main text as well as the figure legends. When comparing biological activity between proteins significant differences are indicated in the figure by asterisks.
